# Plasma biomarkers for prediction of early tumor recurrence after resection of pancreatic ductal adenocarcinoma

**DOI:** 10.1038/s41598-021-86779-x

**Published:** 2021-04-05

**Authors:** Marie-Claire Rittmann, Saskia Hussung, Lukas M. Braun, Rhena F. U. Klar, Esther A. Biesel, Stefan Fichtner-Feigl, Ralph Fritsch, Uwe A. Wittel, Dietrich A. Ruess

**Affiliations:** 1 Department of General and Visceral Surgery, Medical Center and Faculty of Medicine, University of Freiburg, Hugstetter Str. 55, 79106 Freiburg, Germany; 2 Department of Medicine I, Medical Center and Faculty of Medicine, University of Freiburg, Freiburg, Germany; 3 Department of Medical Oncology and Hematology, Zurich University Hospital, Zurich, Switzerland

**Keywords:** Cancer, Molecular biology, Biomarkers, Molecular medicine, Oncology

## Abstract

Pancreatic ductal adenocarcinoma (PDAC) is a disease with a very unfavorable prognosis. Surgical resection represents the only potentially curative treatment option, but recurrence after complete resection is almost certain. In an exploratory attempt we here aimed at identifying preoperative plasma protein biomarkers with the potential to predict early recurrence after resection of PDAC. Peripheral blood samples from 14 PDAC patients divided into three groups according to their time to tumor recurrence after curatively intended resection (early: < 6 months, medium: 6–12 months, late: > 12 months) underwent targeted proteome analysis. Proteins most strongly discriminating early and late recurrence were then examined in a number of established PDAC cell lines and their culture supernatants. Finally, PDAC organoid lines from primary tumors of patients with early and late recurrence were analyzed for confirmation and validation of results. In total, 23 proteins showed differential abundance in perioperative plasma from PDAC patients with early recurrence when compared to patients with late recurrence. Following confirmation of expression on a transcriptional and translational level in PDAC cell lines we further focused on three upregulated (MAEA, NT5E, AZU1) and two downregulated proteins (ATP6AP2, MICA). Increased expression of NT5E was confirmed in a subset of PDAC organoid cultures from tumors with early recurrence. MICA expression was heterogeneous and ATP6AP2 levels were very similar in both organoids from early and late recurrent tumors. Most strikingly, we observed high MAEA expression in all tested PDAC (n = 7) compared to a non-cancer ductal organoid line. MAEA also demonstrated potential to discriminate early recurrence from late recurrence PDAC organoids. Our study suggests that identification of plasma protein biomarkers released by tumor cells may be feasible and of value to predict the clinical course of patients. Prediction of recurrence dynamics would help to stratify up-front resectable PDAC patients for neoadjuvant chemotherapy approaches in an individualized fashion. Here, MAEA and NT5E were the most promising candidates for further evaluation.

## Introduction

Pancreatic ductal adenocarcinoma (PDAC) is an aggressive malignant disease of the exocrine pancreas with a 5-year survival rate less than 10%^1^. Rapid disease progression with early metastatic spread and lack of early clinical symptoms contribute to the poor prognosis. The only curative therapy for PDAC is surgical resection, but only 15 to 20% of all patients are eligible^2^. Most patients suffer from locally advanced or metastatic disease at the time of diagnosis, which precludes surgical options and limits the estimated overall survival of these patients to less than a year^3^.

Almost all patients encounter tumor recurrence after histologically complete tumor resection. Adjuvant chemotherapy is recommended with modified FOLFIRINOX and has been shown to substantially increase overall survival^4^. In patients with lower performance status, gemcitabine + capecitabine^5^, gemcitabine or 5-fluoruracil (5-FU) remains the current standard of care^6,7^. Recurrence occurs quite early after tumor resection and despite adjuvant chemotherapy 30–50% of patients develop a postoperative tumor relapse within one year after surgery^8^. To explain the early postoperative recurrence (up to 6 months after resection), an already disseminated disease is likely, which is not detectable macroscopically at the time of diagnosis or operation. Other studies found lymph node metastasis^9^, perineural invasion^10^ and status of resection margin^11^ to be strongly associated with early tumor recurrence and poor survival outcomes after surgical intervention.

Until today, no biomarker is available to predict prognosis. Even specific and sensitive diagnostic markers for early detection of PDAC are limited. However, carbohydrate antigen Ca19-9 is used in clinical practice for assessment of PDAC progression and monitoring of disease activity^12^. With reliable data on neoadjuvant therapy strategies becoming increasingly available^13^, it becomes more and more essential to identify preoperatively detectable parameters, as patients with resectable tumors with increased risk of early recurrence may benefit from other treatment options such as neoadjuvant chemotherapy before surgery.

In this explorative study we aimed at identifying and characterizing plasma biomarkers that may preoperatively predict PDAC recurrence after curative resection.

## Methods

### Study design, data management and ethics statement

Blood and tissue samples as well as clinical data were collected after having acquired written informed consent from all patients prior to surgery. The study was approved by the Ethics Committee of the University of Freiburg (No. 126/17 and 73/18) and was registered in the German Register of Clinical Studies (DRKS-ID: DRKS00023929; date of registration: 12/17/2020). All methods were performed in accordance with the Declaration of Helsinki and with all relevant guidelines and regulations. Clinical data was continuously documented and analyzed retrospectively. PDAC diagnosis was confirmed by routine pathology after resection of pancreatic tumor.

### Plasma samples

In total, plasma of 14 patients was used in this study. Plasma samples were pooled on the basis of patients’ tumor relapse time (early recurrence: within 6 months after resection, n = 5; medium recurrence: within 6–12 months after resection, n = 4; late recurrence: no relapse 12 months after resection, n = 5). Plasma of PDAC patients was collected one day prior to planned resection and during resection and was analyzed. The plasma samples were evaluated by a plasma proteome array for 1012 pre-defined biomarkers in the panels cardiometabolic, cardiovascular II + III, cell regulation, development, immune response, inflammation, metabolism, neurology, oncology II and organ damage (Olink Bioscience, Uppsala, Sweden). The data were normalized using an internal and interplate control to minimize both intra- and inter-assay variation and then transformed using a pre-determined correction factor. Pre-processed data are provided in the arbitrary unit normalized protein expression (NPX) on a log2 scale. A high NPX value corresponds to a high protein concentration and expresses relative quantification between samples, therefore no comparison of absolute levels between different proteins can be made.

### Cell culture

The pancreatic cancer cell lines PANC-1 (CRL-1469™), MIA PaCa-2 (CRM-CRL-1420™), Capan-2 (HTB-80™) and HPAF-II (CRL-1997™) were obtained from the American Type Culture Collection (ATCC). The human pancreatic stellate cell line EP1077 was established from pancreatic tumor tissue in our department. All cells were kept at 37 °C in a humidified 5% CO_2_ atmosphere using DMEM culture medium supplemented with 10% fetal bovine serum (Gibco) and 1% penicillin/streptomycin (Sigma-Aldrich).

### Patient-derived pancreas organoid (PDO) cultures

Establishment of organoid cultures^14,15^ and determination of *KRAS* mutational status^16^ was achieved as previously described. For passaging, Matrigel domes with organoids were disrupted from a 24-well plate and collected in 10 mL cold splitting medium. Splitting Medium for patient-derived pancreatic ductal organoids was prepared with Advanced DMEM/F-12 medium supplemented with 10 mM HEPES (Gibco) and 1 × GlutaMAX (Gibco). The supernatant was discarded after centrifugation (1200 rpm, 5 min, 4 °C) and the pellet was resuspended in 1 mL TrypLE Express (RT, Gibco) and incubated for 15 min at 35 °C at 180 rpm. Digestion was stopped by adding 10 mL cold splitting medium and followed by centrifugation (1200 rpm, 5 min, 4 °C). The pellet was resuspended in ice-cold Matrigel (Corning) and plated at 1:2 ratio into a 24-well plate with 25 µL Matrigel for each dome. Organoid feeding medium was added after 15 min incubation at 37 °C. Feeding medium consisted of Wnt3a-conditioned medium (50% v/v) containing 500 nM A83-01, 50 ng/mL mEGF, 100 ng/mL hFGF10, 0.01 µM Gastrin I, 1.25 mM N-Acetylcysteine, 10 mM Nicotinamide, 1 × B-27 supplement and 1 × R-Spondin I-conditioned medium (10% v/v). Shortly before use 10.5 µM Rho-associated kinase (ROCK) inhibitor Y-27632 was added to the medium. Organoid feeding medium was changed every 2–3 days and cultures were split once every 7–10 days. All steps were performed on ice, unless indicated differently. For the present analyses organoids were cultured for 2–5 months; passage numbers used for experiments are documented in Table 2.

### RNA-isolation and quantitative PCR (qPCR) analysis

Matrigel domes were disrupted in 1 mL ice-cold PBS for RNA-Isolation. Organoids were centrifuged (5300 rpm, 5 min, 4 °C) and the supernatant discarded. Pellets were resuspended in 500 µL cell recovery solution (Corning) and incubated for 60 min on ice. Finally, pellets were washed with 500 µL ice-cold PBS. After centrifugation (5300 rpm, 10 min, 4 °C), the supernatant was discarded and the organoid pellets were frozen at -80 °C until further analysis.

Total RNA was isolated from organoid cultures and cell cultures using the RNeasy Mini Kit (Qiagen) according to manufacturer’s instructions. RNA concentration and purity was determined using a TECAN Reader infinite M200. For cDNA synthesis, RevertAid First Strand cDNA Synthesis Kit (Thermo Fisher) was used according to manufacturer’s instructions and the cDNA was diluted to 3 ng/µL for qPCR analysis.

qPCR was performed in triplicates and negative controls lacking cDNA were included. Each reaction contained 9 ng of cDNA or noRT-control in a final volume of 10 µL, 5 µL Power SYBR Green PCR Master Mix (Applied Biosystems) and the primer’s concentration was 7.5 µM. The qPCR run on a LightCycler 480 Instrument denature II (Roche) and was as following: (1) hot start cycle at 95 °C for 10 min, (2) 40 cycles at 95 °C for 15 s and (3) annealing at 60 °C for 1 min. Relative gene expression of three independent experiments was normalized to β-actin and calculated by the 2^-ΔΔCt^ method compared to the mean value across all cell lines or to PDO pancreatitis control. The primer sequences were designed as follows:

**Table:** 

NT5E	fwd 5′-GAAGGCCTTTGAGCATAGCG 3′; rev 5′-CGACACTTGGTGCAAAGAACA-3’
AZU1	fwd 5′-GCGTGACGATACTGCCACT-3′; rev 5′-TCACGTTGACAAACCTGGGA-3’
ATP6AP2	fwd 5′-GCCTATACCAGGAGAGCGGA-3′; rev 5′-CCGAGGACGATGAAACAGGT-3’
IFNLR1	fwd 5′-CGTGTACCTGACATGGCTCC-3′; rev 5′-TGGTTCCCGCACACTCTTC-3’
CTSO	fwd 5′-ACAGCATCACTGCTCTAGTGG-3′; rev 5′-ACTCCCCAAGAACTTCCCCA-3’
MICA	fwd 5′-AGAATCCGGCGTAGTCCTGAG-3; rev 5′-ATTCCGGGGATAGAAGCTGGAA-3’
CDCP1	fwd 5′-GAACTGCGGGGTCTCTATCG-3′; rev 5′-CTCGTGGCAGAGCAATCTCA-3’
TNFRSF12A	fwd 5′-TTTTGGTCTGGAGACGATGCC-3′; rev 5′-TCACTGGATCAGCGCCACAG-3’
MAEA	Hs_MAEA_1_SG QuantiTect Primer Assay (Qiagen)

### Immunocytofluorescence staining

Cells were seeded on glass cover slips in DMEM medium and cultured for 48 h. Culture media was removed and cells were fixed with ice-cold methanol for 15 min at − 20 °C and washed with PBS. Fixed cells were permeabilized with 0.5% Triton X-100 (RT) for 5 min. Unspecific binding was blocked with 2% BSA for 30 min at RT and cells were incubated over night at 4 °C with primary antibodies diluted in 2% BSA (rabbit-α-NT5E, Cell Signaling 1:75; rabbit-α-AZU1, LSBio, 1:250; rabbit-α-ATP6AP2, LSBio, 1:75; goat-α-MICA, R&D Systems, 1:250; mouse-α-MAEA, R&D Systems, 1:250). After washing with PBS, the cells were incubated for 60 min with the respective secondary antibodies (goat-α-rabbit Cy3, Thermo Fisher 1:250; monkey-α-goat Alexa 594, Abcam 1:500; goat-α-mouse Cy3, Invitrogen 1:250). After washing with PBS the cells were stained with DAPI (1:10.000 in aqua dest.) for 2 min. Afterwards, the coverslips were embedded in Kaiser's glycerol gelatine. Samples were analyzed with a fluorescence microscope (Olympus).

### Immunohistochemistry (IHC) staining

For IHC staining, organoid-domes were fixed in the culture plate with 500 µL 4% paraformaldehyde (PFA) for 15 min. Removal of PFA was followed by embedding the domes in 1 mL 2% agarose gel. Samples were then dehydrated and embedded in paraffin, sectioned at 3 µm and placed on slides. The sections were deparaffinized using ROTIHistol (Roth) and rehydrated in an alcohol gradient. Antigen retrieval was performed in a pressure cooker for 15 min with 10 mM sodium citrate (pH 6.0 with 0.05% Tween20). Endogenous peroxidase was quenched with 3% H_2_O_2_ for 30 min, followed by three washing steps with TBS-T (pH 7.6, 0.05% Tween20). Non-specific background was blocked with 1% BSA/TBS-T for 60 min. The primary antibodies were diluted in 1% BSA/TBS-T (rabbit-α-NT5E, Cell Signaling, 1:250; rabbit-α-AZU1, LSBio, 1:1500; rabbit-α-ATP6AP2, LSBio, 1:1000; rabbit-α-MICA, Abcam, 1:1000; mouse-α-MAEA, R&D Systems, 1:50) and incubated at 4 °C overnight. Slides were washed three times in TBS-T and incubated with EnVision anti-rabbit- or anti-mouse-HRP (DAKO) for 60 min at RT. Slides were again washed three times in TBS-T. Immunoreactions were detected using DAB (1:50) as chromogen for 2 min, followed by a washing step in water. Finally, the slides were counterstained with Mayer’s hematoxylin (1:5 in aqua dest.) for 5 min, followed by incubation in warm water for 10 min. Slides were dehydrated with graded alcohols, cleared in Xylene and mounted using the ROTIHistokitt II (Roth).

### Western Blot

Total protein extracts were prepared from PDAC cell lines PANC-1, MIA PaCa-2, HPAF-II and Capan-2 and the stellate cell line EP1077 by lysis in RIPA-buffer (50 nM Tris, 150 nM NaCl, 1% Nonidet P-40, 0.5% Sodium Deoxycholate, 1 mM EDTA, 0.1% SDS) supplemented with protease inhibitors (complete protease inhibitor cocktail, Roche). Protein concentrations were determined at 562 nm on the TECAN Reader infinite M200. Equal amounts of the respective cell lysates (20 μg total protein) were separated after denaturation (95 °C, 5 min) on a 12% SDS-PA gel (60 min, 150 V). Afterwards the gel was blotted in Towbin transfer buffer (20% methanol) onto a nitrocellulose membrane (120 min, 300 mA). The membrane was blocked with 5% milk powder (MP)/TBS-T for 60 min at RT. Overnight incubation with the primary antibodies was performed at 4 °C with orbital shaking (NT5E (Cell Signaling, 1:2000 5% BSA/TBS-T), AZU1 (LSBio, 1:500 5% MP/TBS-T), ATP6AP2 (LSBio, 1:500 5% MP/TBS-T), MICA (Abcam, 1:1000 5% MP/TBS-T) and MAEA (R&D Systems, 1:750 5% BSA/TBS-T)). The membrane was washed with TBS-T and subsequently incubated with horseradish peroxidase (HRP)-conjugated secondary antibodies (α-rabbit HRP, Cell Signaling; α-mouse HRP, Cell Signaling) at a dilution of 1:5000 in 5% MP/TBS-T or 5% BSA/TBS-T for 90 min at RT on the shaker. The membrane was washed with TBS-T and the signal amplified with a chemiluminescent reagent (Thermo Fisher Scientific) before the membrane was developed using the BioRad ChemiDoc system. For loading control, the membrane was stripped with TBS-T for 60 min and incubated with the α-PDI antibody (Enzo Life Sciences, 1:1000) in 5% MP/TBS-T for 90 min at RT. The signal was again detected after incubation with HRP-conjugated secondary antibodies (α-rabbit HRP, Cell Signaling).

### Enzyme-linked immunosorbent assay (ELISA)

Levels of respective proteins were quantified with Human MICA ELISA Kit (LS-Bio, LS-F150), Human ATP6AP2 ELISA Kit (Aviva Systems Biology, OKEH04445), Human NT5E ELISA Kit (Biorbyt, orb385324) and Human AZU1 ELISA Kit (Biorbyt, orb390828). All incubations were performed in a closed chamber at room temperature and according to the manufacturer’s instructions. Wells were precoated with target specific antibodies. 100 μL of standard, blank and cell culture supernatants were added in duplicates and incubated for 150 min with constant shaking. The plate was then washed four times with washing buffer. 100 μL of detection antibody solution was added, followed by 60 min incubation with gentle shaking. The wells were emptied and washed four times with wash solution. HRP-Streptavidin solution (100 µL) was incubated for 45 min with constant shaking. Finally, 10 μL Tetramethylbenzidine (TMB) substrate was added and incubated for 30 min with constant shaking. The optical density was determined at 450 nm using the TECAN Reader Infinite M200 after addition of 50 μL Stop Solution.

### Statistical analysis and software

Each experiment was performed at least three times independently, unless otherwise stated. All statistical evaluations were performed with the statistical software GraphPad® Prism version 8.4 or Microsoft® Excel 2016. Differences between the measured values in the ELISAs were investigated with one-way analysis of variance (ANOVA) followed by Tukey’s post-hoc test. Data obtained from organoid experiments was analyzed using Student’s t-test following testing for normal distribution based on Shapiro–Wilk. The average values are given as mean value ± standard error of the mean (SEM). P-values of < 0.05 were considered statistically significant. Significances were shown with symbols (*p < 0.05; **p < 0.01; ***p < 0.001). Figures were created using GraphPad® Prism version 8.4 and Microsoft® Excel 2016 and PowerPoint 2016.

## Results

### Plasma samples show differences in protein levels between PDAC patients with early and late recurrence

**Figure 1:**
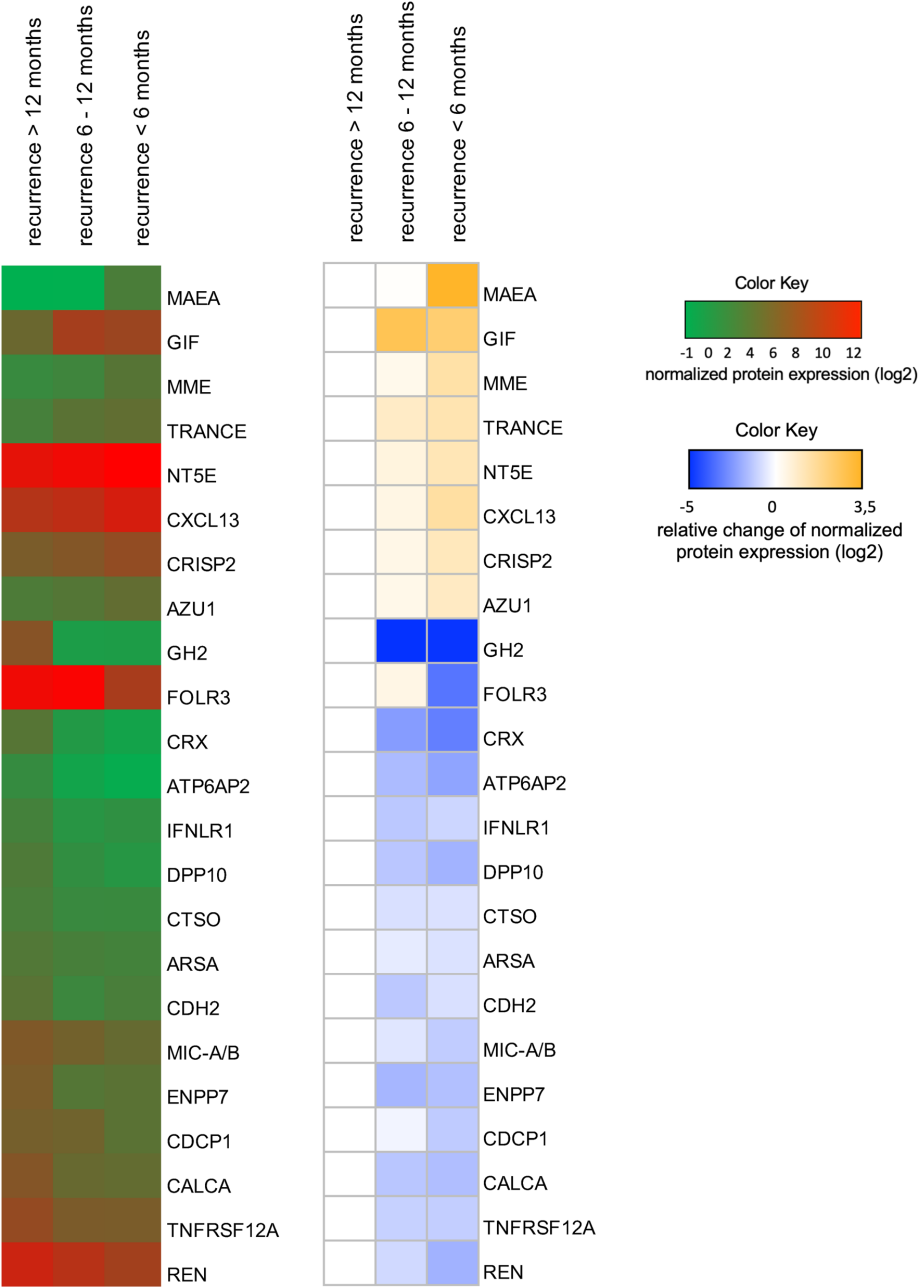
Normalized protein expression of plasma samples from PDAC patients with early (< 6 months), medium (6–12 months) and late (> 12 months) recurrence times. Pooled samples of 4–5 patients per recurrence time were analyzed. The samples were taken one day prior (preoperative sample) and directly after the surgical removal of the specimen. The values for preoperative and intraoperative sample were summarized for the respective recurrence times, since only GH2 showed an influence of the operation on the protein level. The 23 proteins displayed showed at least a twofold change in protein expression between early and late recurrence. Left: Heat map of absolute values, displayed as log2. Right: Heat map of relative values as fold-change in comparison to late recurrence, set to 1, also displayed as log2.

In order to identify potential tumor biomarkers projecting early postoperative recurrence, pooled plasma samples from PDAC patients with different recurrence times after curatively intended pancreatic surgery were tested in a targeted proteome analysis approach using a plasma proteome array for 1012 proteins (Olink Bioscience, Uppsala, Sweden). Plasma from a total of 14 patients diagnosed with PDAC was collected just before and during surgery. Samples were pooled into three groups according to time until tumor recurrence: Five patients experienced a relapse within 6 months after resection (termed early recurrence), 4 patients had a relapse 6–12 months after resection (termed medium recurrence) and in 5 patients no tumor could be detected even 12 months after surgery (termed late recurrence). Clinicopathological patient characteristics are summarized in Table 1. The values for preoperative and intraoperative samples were summarized for the respective recurrence times, since only one single protein (GH2) showed an influence of the operation on expression level (data not shown). Twenty-three potential biomarker proteins were then selected for further analysis based on their at least twofold change in expression levels between early and late recurrence samples (Fig. 1). Interestingly, for the majority of markers, the results for samples from patients with medium recurrence placed between those from patients with early and late recurrence. This however limited their comparative evaluation in both directions. We therefore decided to focus on the comparison only between early and late recurrence for the subsequent analyses. Eight of the 23 selected proteins demonstrated relatively higher abundance in plasma from patients with early recurrence compared to patients with late recurrence. Particularly relevant were MAEA and GIF, which showed 12-fold and fivefold higher concentrations respectively in patients with early recurrence. Fifteen proteins displayed lower abundance in plasma from patients with early recurrence. The protein levels for GH2 in patients with early recurrence were 33-fold lower compared to those in patients with late recurrence, with probable bias due to the above-mentioned observation. A fivefold to 11-fold decrease in early vs. late recurrence samples was observed for ATP6AP2, FOLR3 and CRX.

**Table 1:** Clinicopathological data of patients who provided plasma samples analyzed in this study.

Pooled sample	Sex	Age	Entity	Grading	UICC Stage and R-Status	Chemo-Tx	Recurrence
EARLY	m	59y	PDAC	G2	pT3, pN0 (0/18). L0. V0. Pn1. R0	Adjuvant Gem + Cap	Early (L + D)
	f	81y	PDAC	G3	pT3, pN1 (2/20). L1. V0. Pn1. R1	None (postop complic.)	Early (L)
	f	55y	PDAC	G2	pT3, pN1 (2/17). pM1(hep). R0 loc	Additive FOLFIRINOX	Early (D)
	f	74y	PDAC	G2	pT3, pN0 (0/14). L0. V0. Pn1. R0	Palliative Gem + Ab	Early (L + D)
	m	76y	PDAC	G3	pT3, pN2 (14/22). L1. V0. Pn1. R0	Palliative Gem + Ab	Early (D)
MEDIUM	f	57y	PDAC	G3	pT3, pN2 (16/28). L1. V0. Pn1. R0	Adjuvant Gem (rec.)	Medium (d.n.a.)
	f	59y	PDAC	G3	pT3, pN2 (5/16). L1. V0. Pn1. R0	Adjuvant Gem	Medium (L + D)
	m	80y	PDAC	G3	pT3, pN1 (2/16). L1. V0. Pn1. R0	Adjuvant Gem	Medium (L)
	f	57y	PDAC	G2	pT3, pN2 (4/26). L1. V0. Pn1. R0	Adjuvant Gem	Medium (L)
LATE	f	76y	PDAC	G2	pT2, pN1 (2/34). L1. V0. Pn0. R0	Adjuvant Gem	Late (none at 44mo. of FU)
	m	73y	PDAC	G2	pT3, pN0 (0/12). L0. V0. Pn1. R0	Adjuvant Gem + Cap	Late (L at 33mo. of FU)
	m	65y	PDAC	G2	pT3, pN1 (1/18). L0. V0. Pn1. R0	Adjuvant Gem + Cap	Late (none at 48mo. of FU)
	f	77y	PDAC	G2	pT3, pN0 (0/20). L0. V0. Pn1. R0	Adjuvant Gem	Late (none at 57mo. of FU)
	m	72y	PDAC	G2	pT3, pN0 (0/12). L0. V0. Pn1. R0	Adjuvant Gem	Late (none at 56mo. of FU)

*d.n.a.*: data not available; *Gem*: gemcitabine; *Ab*: nab-Paclitaxel; *Cap*: Capecitabine; *Early*: tumor recurrence within the first 6 months after curatively intended resection; *Medium*: tumor recurrence within 6–12 months after resection; *Late*: no tumor recurrence even 12 months after resection; *L*: local recurrence, *D*: distant recurrence (hepatic/pulmonal/peritoneal carcinomatosis); *FU*: follow-up.

### Expression analysis of potential biomarkers in established pancreatic cancer cell lines

**Figure 2:**
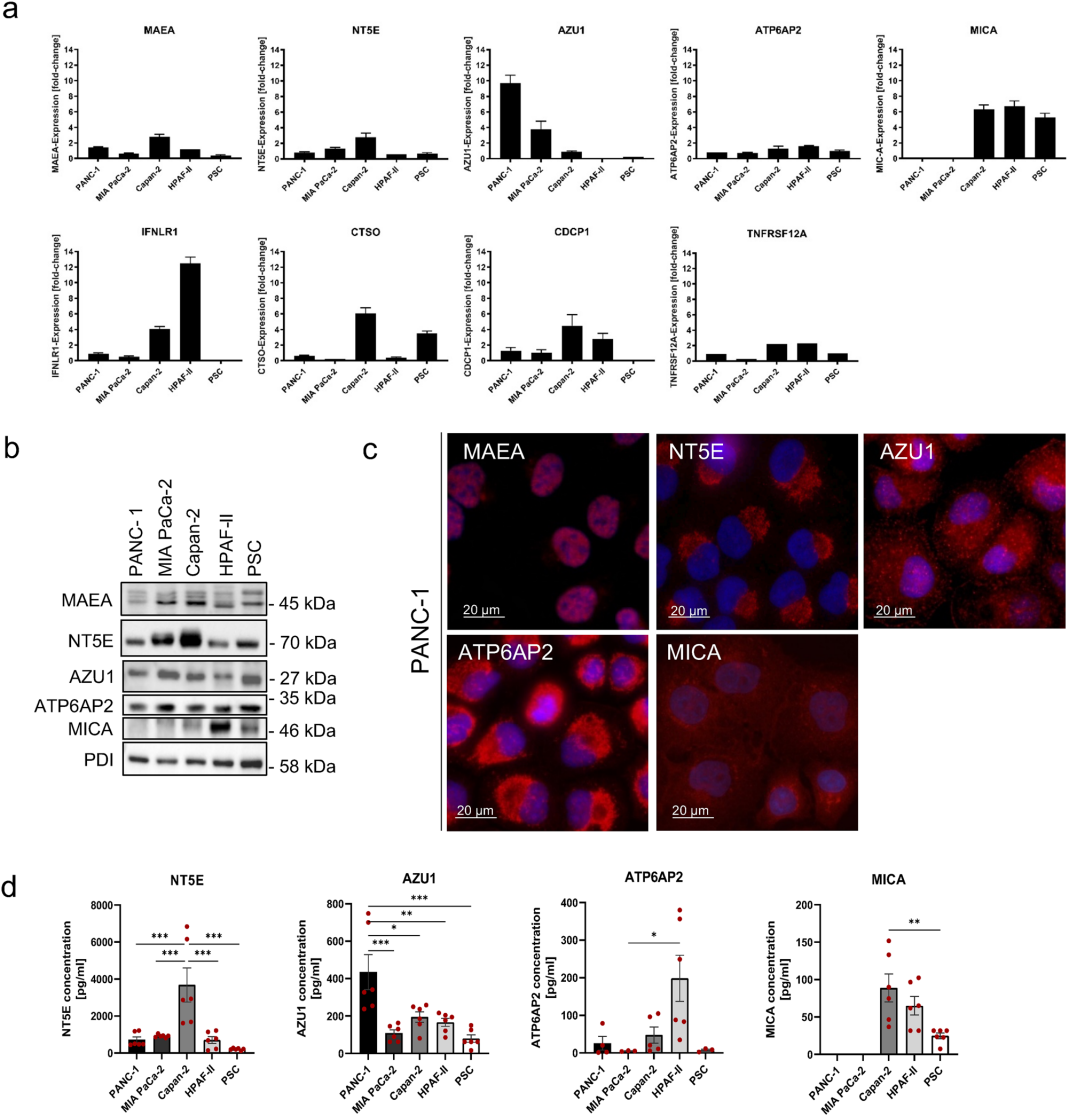
Characterization of proteins in pancreatic cancer cell lines (PANC-1, MIA PaCa-2, Capan-2, HPAF-II) and pancreatic stellate cell line (PSC). (**A**) The mRNA levels were determined by qPCR for the genes MAEA, NT5E, AZU1, ATP6AP2, MICA, IFNLR1, CTSO, CDCP1 and TNFRSF12A. The expression level of each gene was normalized against β-actin. Data is presented as mean ± SEM (n = 3). (**B**) Expression of MAEA, NT5E, AZU1, ATP6AP2 and MICA was detected by Western blot analysis. Representative Western blots were selected from three independent experiments. PDI was included as a loading control. Full membrane blots are provided in Supplementary Figure S1. (**C**) Representative immunofluorescence images of PANC-1 cells stained for MAEA, NT5E, AZU1, ATP6AP2 and MICA (red). Nuclei are stained in blue with DAPI. (**D**) Protein concentrations of NT5E, AZU1, ATP6AP2 and MICA in cell culture supernatants were determined by sandwich ELISAs. For values outside the linear range, the value of the smallest standard as minimum or the value of the largest standard as maximum was used for presentation. The mean value ± SEM is given (n = 3 in duplicates). Statistical evaluation was performed by one-way ANOVA + Tukey’s post-hoc test): *p < 0.05; **p < 0.01; ***p < 0.001.

To further validate our findings from patient plasma samples and to confirm a link to expression by PDAC cells, we analyzed mRNA expression of our 23 target genes in four established PDAC cell lines and one human pancreatic stellate cell line (PSC) as a control by qPCR. Nine of the 23 genes were expressed in all tested PDAC lines (Fig. 2A). We could observe that two of these genes, ATP6AP2 and TNFRSF12A, were expressed similarly and on low levels in all cell lines investigated. The other seven genes demonstrated differential expression across the four tumor cell lines. NT5E expression was found to be the highest in Capan-2, the same applied for MAEA, CDCP1 and CTSO. The expression of these markers in Capan-2 was 3- to 6-times higher than the mean value over all cell lines. Interestingly, other genes like AZU1 showed a very strong expression in PANC-1 followed by MiaPaCa-2, with a nearly 10- and 4-times higher expression respectively and poor signals in Capan-2 and HPAF-II. The opposite expression pattern was seen with MICA, demonstrating strong expression in Capan-2 and HPAF-II, but weak signals in PANC-1 and MIAPaCa-2. The same was found for IFNLR1, but with 12.5 times higher expression in HPAF-II. To discriminate expression by cancer cell lines and tumor stroma myofibroblasts, a pancreatic stellate cell line was also evaluated. Strong expression in PSC’s was only seen for MICA and CTSO, but not for the other genes (Fig. 2A).

Based on both, proteome array results and available literature on the relevance for pancreatic cancer, confirmatory analyses were performed for a selection of five genes and their corresponding proteins. We performed Western blot for AZU1, ATP6AP2, MAEA, MICA and NT5E with lysates obtained from the four PDAC cell lines and from our PSC cell line as control (Fig. 2B). MAEA showed the highest protein levels in Capan-2, followed by MIAPaCa-2 and PSC. Lower protein levels were detected in PANC-1 and HPAF-II. Consistent with mRNA levels, NT5E was present in all tumor cell lines and the stellate cell line with higher protein levels in Capan-2 and MIAPaCa-2. AZU1 and ATP6AP2 were also expressed in all cell lines. Interestingly, no large differences in protein expression could be observed in the Western blot while qPCR data indicates strong differences in AZU1 expression. Protein levels for MICA were highest in HPAF-II, followed by PSC and Capan-2, in line with the observations on mRNA level (Fig. 2B). Immunofluorescence staining revealed that the MAEA protein was located in the nucleus of PANC-1 cells, whereas NT5E, ATP6AP2 and MICA were mainly localized in the cytoplasm; AZU1 was also localized in the cytoplasm but was predominantly found in vesicles (Fig. 2C).

To confirm the release of our target proteins from pancreatic cancer cells we evaluated the concentrations of candidate biomarkers in the cell culture supernatant (Fig. 2D). NT5E concentration was highest in Capan-2 supernatant with lower concentrations in the other cell lines. AZU1 had the highest concentration in PANC-1 supernatant, in line with the qPCR results. The other cell lines displayed lower values. Concentration of ATP6AP2 was low in supernatants from all cell lines, except for HPAF-II, in spite of rather comparable expression across cell lines as determined by qPCR and Western blot. MICA was not detectable in PANC-1 and MIAPaCa-2 supernatants, in consistency with the low levels on mRNA and whole cell lysate protein level.

### PDO cultures confirm differential expression between early and late recurrence samples for selected biomarker candidates

As an additional step of validation for our findings from the proteome array and cell line analysis we established patient-derived organoid cultures (PDO), which can be generated directly from small PDAC tissue biopsies. This allowed modeling of individual tumor biology stratified by recurrence patterns in vitro. Organoids were retrospectively classified into two groups, according to the clinical course of corresponding patients. In total we analyzed seven pancreatic ductal organoid cultures of patients with known time points of tumor recurrence and clinical characteristics as given in Table 2. In 4 patients recurrence occurred within 6 months after resection (termed early recurrence; organoid lines E1-E4) and 3 patients experienced no relapse within the first 12 months after resection (termed late recurrence; organoid lines L1-L3). A ductal organoid culture from a patient with chronic pancreatitis served as control (ctrl).

**Table 2:** Clinicopathological data of patients who provided tissue for patient-derived organoid cultures (PDO) used in this study.

Organoid ID (passage #)	Sex	Age	Entity	Grading	UICC Stage and R-Status	KRAS mutation	Adjuv./Addit. Tx	Recurrence
E1 (P15-P16)	m	35y	PDAC*	G2	ypT3, pN1 (3/14). L0. V0. Pn1. R1	KRAS G12R	Additive RT; Gem + Ab	Early (L + D)
E2 (P14-P15)	m	70y	PDAC	G2	pT3, pN2 (7/27). L1. V0. Pn1. R1	KRAS G12D	Adjuvant Gem + Cap	Early (D)
E3 (P18-P20)	f	68y	PDAC	G2	pT3, pN2 (5/37). L1. V1. Pn1. R0	KRAS G12D	None (postop complic.)	Early (L + D)
E4 (P21-P22)	f	60y	PDAC	G2	pT3, pN0 (0/7). L0. V1. Pn1. R0	KRAS G12D	d.n.a	Early (d.n.a.)
L1 (P15-P21)	m	79y	PDAC	G3	pT3, pN2 (6/16). L1. V0. Pn1. R0	KRAS G12R	Adjuvant Gem + Cap	Late (L + D at 18mo. of FU)
L2 (P10-P21)	m	73y	PDAC	G3	pT2, pN2 (4/22). L1. V0. Pn1. R0	KRAS G12D	Adjuvant Gem + Cap	Late (none at 24mo. of FU)
L3 (P6-P8)	m	59y	AdenoCa	G2	pT2, pN1 (1/19). L1. V0. Pn0. R1	KRAS WT	d.n.a	Late (none at 12mo. of FU)
Ctrl (P10-P11)	m	63y	Chronic pancreatitis	n.a	n.a	KRAS WT	n.a	n.a

*n.a.*: not applicable; *d.n.a.*: data not available.

*Neoadjuvant therapy w/ 3 cycles FOLFIRINOX and insufficient response; *RT*: radiotherapy; *Gem*: gemcitabine; *Ab*: nab-Paclitaxel; *Cap*: capecitabine; *Early*: tumor recurrence within the first 6 months after curatively intended resection; *Late*: no tumor recurrence even 12 months after resection; *L*: local recurrence, *D*: distant recurrence (hepatic/pulmonal/peritoneal carcinomatosis); *FU*: follow-up.

**Figure 3:**
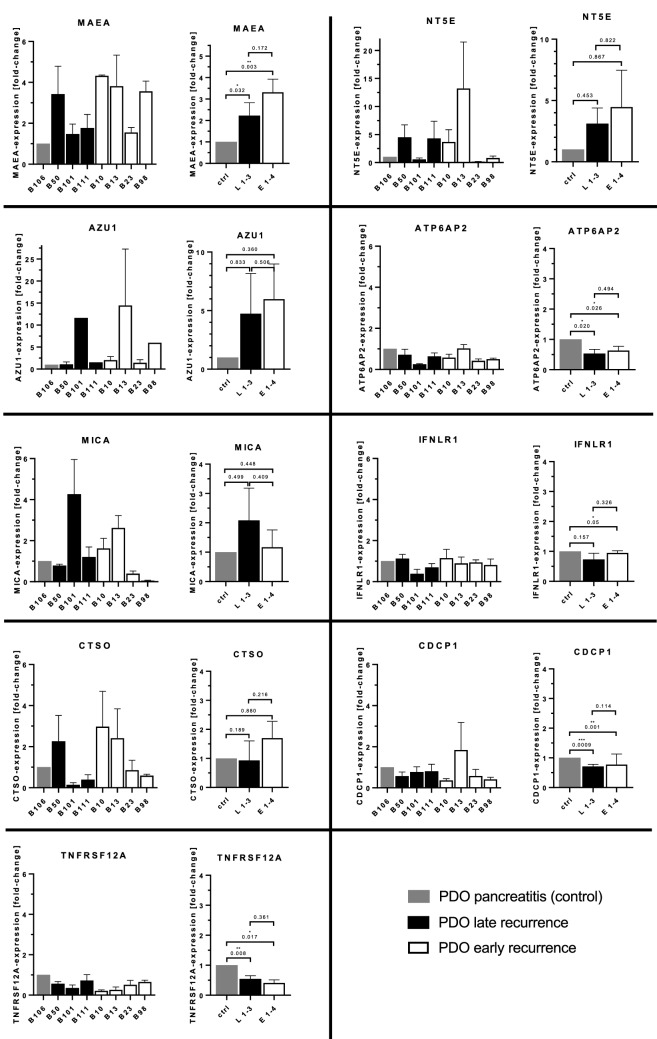
Gene expression analysis of patient-derived PDAC organoid cultures. The mRNA levels in seven organoid cultures (late recurrence: L1, L2, L3/early recurrence: E1, E2, E3, E4) were determined by qPCR for the genes MAEA, NT5E, AZU1, ATP6AP2, MICA, IFNLR1, CTSO, CDCP1 and TNFRSF12A. A ductal organoid line from a chronic pancreatitis patient (ctrl) served as control. The expression level of each gene was normalized against β-actin. Data is presented as mean ± SEM (n = 3), except AZU1 for E4, L2, L3 (n = 1). For each gene of interest, graphs on the left depict results for individual organoid lines, and graphs on the right a merged analysis including statistics. Statistical evaluation was performed using Student’s t-test: *p < 0.05; **p < 0.01; ***p < 0.001.

First, we assessed expression of the previously selected nine genes (as in Fig. 2A) by qPCR in organoid cultures of patients with early and late recurrence (Fig. 3). All of the markers tested were expressed by organoid lines, but in some cases with large differences. It was noticeable that E3 had low expression values for all markers, whereas many markers were highly expressed in E2. MAEA expression was quite consistently upregulated and showed up to 4-times higher expression in PDAC organoid lines compared to the single non-cancer control. It’s mean value appeared about 1.5-times higher in early recurrence compared with late recurrence organoids. Although not statistically significant in direct comparison, this observation was in accordance with the results from the plasma proteome array. In contrast to MAEA, levels of NT5E were much more variable. We saw high expression in two patients with early recurrence (E2, E1) and low values for E3 and E4. These results might indicate an increased NT5E expression in a subgroup of early recurrence cases. AZU1 expression was increased in three PDAC organoid cultures (E2, E4, L2) and at the same level as in the control in the other lines. In summary, elevated AZU1 expression appears to be observable in a subgroup of cases, irrespective of the tumor recurrence time. CTSO expression was elevated in one out of three late recurrence samples but two out of four early recurrence samples. Overall analysis thus suggested a trend for higher expression levels in early recurrence than in late recurrence samples. Results for MICA transcription were heterogeneous, with higher and lower expression levels in samples from both recurrence groups. For ATP6AP2, IFNLR1, CDCP1 and TNFRSF12A expression we observed similarly reduced levels in early and late recurrence PDAC organoids in comparison to the control sample (Fig. 3).

**Figure 4:**
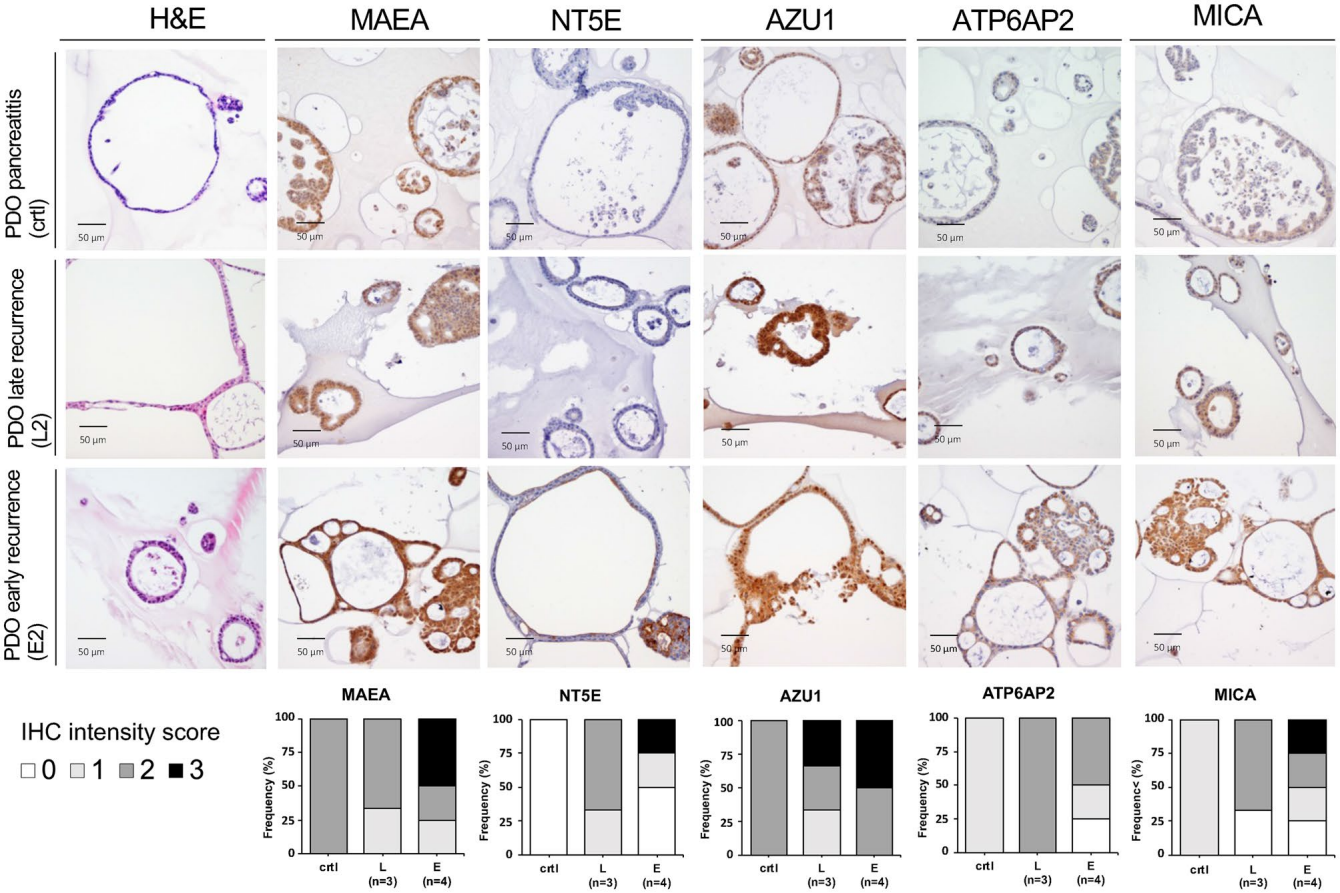
Immunohistochemistry staining of PDAC organoid cultures for MAEA, NT5E, AZU1, ATP6AP2 and MICA. The same organoid cultures as in Fig. 3 were analyzed. Brown color represents a positive staining. Staining intensity was semi-quantitatively scored as: 0 (negative), 1 (weak), 2 (moderate), 3 (strong) and is shown in the bottom panels as stacked bar graphs. For representation we selected organoid culture L2 for late recurrence, E2 for early recurrence and the non-tumor chronic pancreatitis organoid line as control.

Next, to examine protein translation, immunohistochemistry for ATP6AP2, AZU1, MAEA, MICA and NT5E was performed on the PDO cultures. Semiquantitative analyses supported our observations from qPCR, yielding largely corresponding staining patterns. For presentation we selected one representative organoid culture from each recurrence time, E2 for early and L2 for late recurrence (Fig. 4). Of note, the organoids quite reliably recapitulated primary tumor tissue; correlating immunohistochemistry sections of organoid source tissue biopsies are provided in Supplementary Fig. S2.

## Discussion

In an exploratory analysis we here aimed at identifying potential plasma protein biomarker candidates for prediction of early tumor recurrence after curatively intended PDAC resection. Following targeted plasma proteome analysis we were able to confirm expression and secretion of a number of candidates by established pancreatic cancer cell lines with correlation in representative patient-derived PDAC organoids.

Our initial analysis identified 23 proteins with differential abundance in the plasma of patients with early and late recurring pancreatic cancer following resection. We could confirm the transcription of nine genes by qPCR (for a summary of previously reported molecular implications and correlations with PDAC tumor dynamics see Table 3) in four different established, commercially available pancreatic cancer cell lines and further analyzed ATP6AP2, AZU1, MICA and NT5E on the basis of available literature on relevance in PDAC. MAEA was analyzed for its meaningful differential expression levels in our plasma sample analysis. On protein level, expression of MAEA, NT5E and to a great extent of AZU1 and MICA but not of ATP6AP2 was confirmed in the PDAC cell lines.

**Table 3:** Summary of characteristics of potential biomarkers indicating early recurrence of PDAC following resection.

Gene	Localization	Clinical relevance for PDAC	Fold-change (early recurrence)	References
ATP6AP2	PM, ER, Vesicles	Highly expressed in PDAC tissues and PDAC cell lines. Essential for activation of Wnt/ ß-catenin signaling pathway in PDAC cells, promotes PDAC cell proliferation.	− 4.9	^23,24^
AZU1	Vesicles	Detected in pancreatic juice of PDAC patients.	+ 2.0	^25^
CDCP1	PM	Represses epithelial phenotype of pancreatic cancer cells. High expression is correlated with poor prognosis in PDAC.	− 2.5	^33,34^
CTSO	Lysosome	No published data.	− 1.7	–
IFNLR1	Cytosol	No published data.	− 2.0	–
MAEA	Nucleoplasm	No published data.	+ 11.7	–
MICA	Cytosol, PM	MICA expression is an indicator of good prognosis in PDAC. Elevated sMICA levels are associated with a poor survival in patients with PDAC. Poorly differentiated PDAC show high sMICA expression.	− 2.4	^21,35^
NT5E	Cytosol, PM	Overexpressed in many types of cancer. High expression correlates with poor OS and DFS in solid tumors. Upregulated in PDAC and correlated with poor prognosis.	+ 2.3	^17,19,20^
TNFRSF12A	Cytosol, PM	Elevated expression in pancreatic cancer cell lines.	− 2.3	^36^

*PM*: plasma membrane; *OS*: overall survival; *DFS*: disease-free survival.

NT5E is the coding gene for the ecto-5’-nucleotidase (CD73) which hydrolyzes extracellular AMP to adenosine^17^. CD73-generated extracellular adenosine enhances tumor growth, angiogenesis and metastasis and suppresses antitumor immune responses^18^. Increased NT5E expression has previously been associated with poor prognosis and correlated with tumor invasion and metastasis in PDAC and other solid tumors^17,19,20^. In agreement with these data we found that NT5E levels were elevated in the plasma of patients with early recurrence. We could also detect an elevation of NT5E expression in individual PDAC organoids established from tumors with early recurrence.

High MICA (MHC class I polypeptide-related sequence A) levels have been linked to favorable prognosis in PDAC^21^. MICA is a MHC class I related molecule but not involved in peptide presentation. It rather functions as a stress-induced antigen and is recognized by activating NKG2D receptors on NK cells, γδ- and CD8^+^-T-cells, resulting in cell lysis at high expression^22^. In line with this we found lower MICA levels in plasma from patients with early recurrent tumors. Unfortunately, we could not detect or confirm a clear pattern of expression differences comparing organoid lines from tumors with early and late recurrence and non-tumor tissue.

ATP6AP2 encodes the (Pro)renin receptor, which is reported to induce the proliferation of pancreatic cancer cells through Wnt/β-catenin pathways^23^. In previous studies ATP6AP2 was reported to be overexpressed in many cancer types, including pancreatic cancer^23,24^. In contrast, in our case ATP6AP2 levels were lower in blood from patients with early recurrence and its expression appeared to be somewhat lower in PDAC organoids compared to the non-cancer control. We were however not able to substantiate differences between early and late recurrence on the organoid level.

AZU1, which we found to be elevated in the plasma of patients with early recurrence, has previously been detected in the pancreatic juice of PDAC patients^25^. The protein is called Azurocidin-1 or Heparin-binding protein and has antimicrobial activity and chemoattracts monocytes^26^. It is also discussed as a potential biomarker for early diagnosis of sepsis^27^. We found an elevated AZU1 expression only in some PDAC organoid cultures, but from both recurrence time groups. Its function in cancer is largely unknown, and based on our data its relevance for pancreatic cancer progression remains elusive.

Yet, one very promising protein appears to be MAEA. To date, the role of MAEA in cancer remains unclear and no previous studies in pancreatic cancer appear to exist. Even its precise biological function remains controversial. It is reported to be part of the CTLH E3 ubiquitin-protein ligase complex, which promotes cell proliferation, and MAEA is especially required for the catalytic activity of the complex^28,29^. One study describes that the CTLH complex in whole, consisting of eleven proteins, is overexpressed in some types of cancer^30^. MAEA has also been found to mediate the attachment of erythroblasts to macrophages, which promotes terminal maturation and enucleation of erythroblasts, presumably by suppressing apoptosis^31^. In non-erythroid cells MAEA partially co-localizes with actin in the nucleus and undergoes dynamic rearrangements in mitotic cells, suggesting involvement in cytokinesis and cell division^32^. In our experiments MAEA was perioperatively detected in the plasma of pancreatic cancer patients, especially of those with early tumor recurrence after resection. In comparison, the plasma abundance was remarkably lower in patients with medium and late recurring tumors. These results were confirmed by an observable tendency towards higher expression of MAEA on transcriptional and translational level in PDAC organoids of patients with early recurrence, compared with late recurrence. MAEA thus appears to be an interesting candidate for further evaluation.

Taken together, two potential plasma protein biomarker candidates may be further evaluated based on this study: first NT5E, which is of great interest for pancreatic cancer as elevated expression has been connected to a poor prognosis of patients, and second MAEA, which might be involved in cell proliferation and thereby linked to aggressive tumor biology.

The here presented work certainly allows only limited conclusions given the rather small sample size. Additionally, patient selection was not controlled for by balancing pathological examination results, thus differences in histological staging may have introduced selection bias. Next, the primary exploratory proteome analysis was performed only once, which might have resulted in a high rate of marker drop-out. And last, protein expression in organoids may be altered by the required specific culture conditions and by the lack of interaction with microenvironmental cell-types and cues. Thus, further confirmation in a larger cohort of patients and probably the generation and validation of a biomarker panel are required.

Yet, our data demonstrate—in a hypothesis generating approach—that identification of preoperative plasma protein biomarkers with potential to predict early recurrence after PDAC resections may be feasible. Preoperative projection of late or early recurrence based on a simple blood draw would help to better stratify up-front resectable PDAC patients for neoadjuvant chemotherapy in an individualized approach.

## Supplementary Information

Supplementary Information.
