# Supplementary material for: Plasma biomarkers for prediction of early tumor recurrence after resection of pancreatic ductal adenocarcinoma
Source: Sci Rep. 2021 Apr 5;11:7499. doi: 10.1038/s41598-021-86779-x (PMC8021576; doi:10.1038/s41598-021-86779-x)
Supplement: Supplementary file 1 — Supplementary Information. [file 41598_2021_86779_MOESM1_ESM.pdf]

Supplementary Data

to

Plasma biomarkers for prediction of early tumor recurrence after resection of pancreatic ductal adenocarcinoma

by

Marie-Claire Rittmann, Saskia Hussung, Lukas M. Braun, Rhena F.U. Klar, Esther A. Biesel, Stefan Fichtner-Feigl, Ralph Fritsch, Uwe A. Wittel, Dietrich A. Ruess

Supplementary Figure S1. Full-length western blots including protein ladders of data depicted in Figure 2b. Protein expression of MAEA, NT5E, AZU1, ATP6AP2 and MICA in PANC-1, MiaPaCa-2, Capan-2, HPAF-II, PSC and HEK293 cell lines is shown. Western blot analysis was performed as indicated. PDI was used as an internal control. Both shorter (top panel) and longer exposure (bottom panel) of AZU1 of the same blot were shown.

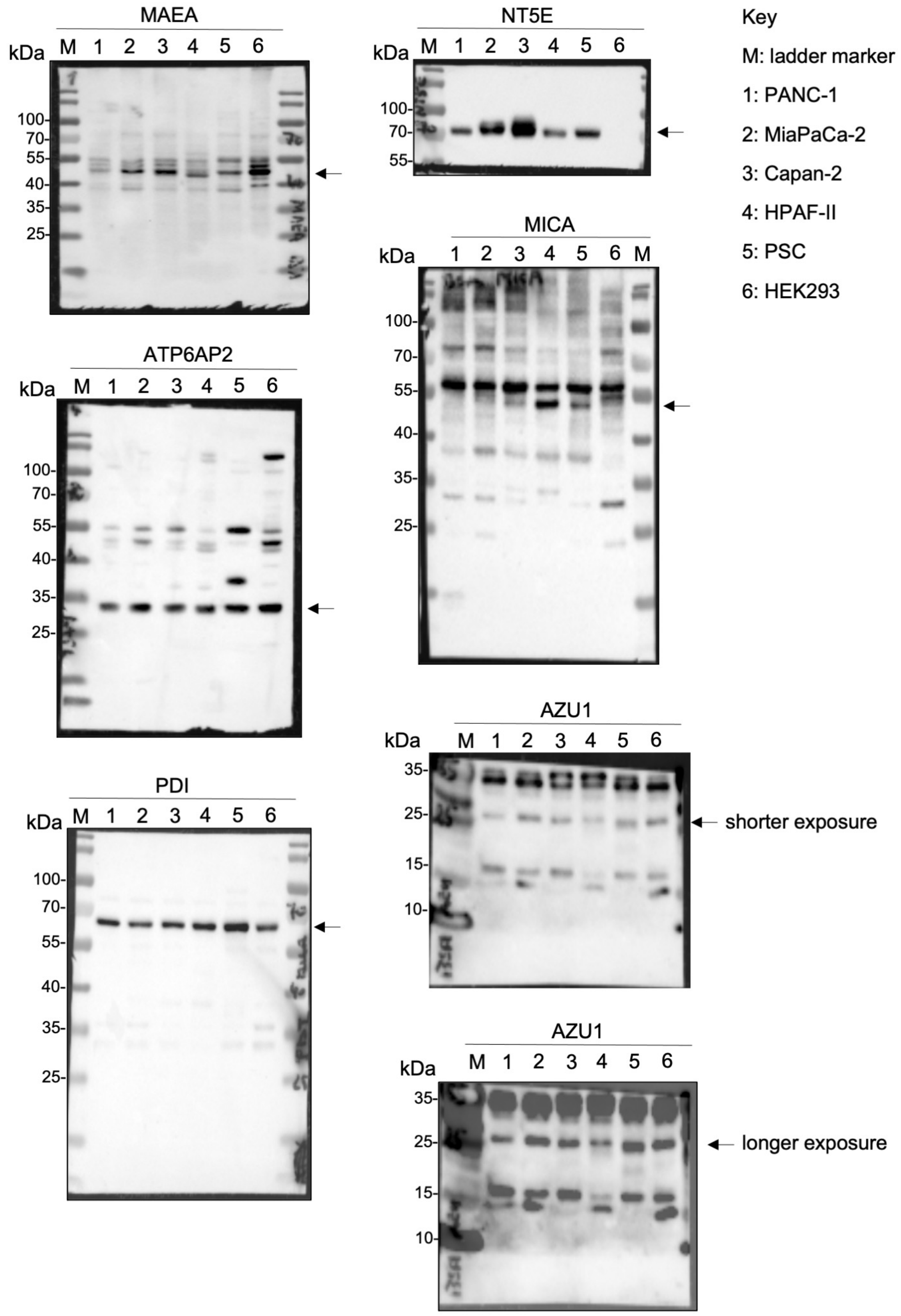

MAEA
kDa M 1 2 3 4 5 6
100-
70-
55-
40-
35-
25-
NT5E
kDa M 1 2 3 4 5 6
100-
70-
55-
Key
M: ladder marker
1: PANC-1
2: MiaPaCa-2
3: Capan-2
4: HPAF-II
5: PSC
6: HEK293
MICA
kDa 1 2 3 4 5 6 M
100-
70-
55-
40-
35-
25-
ATP6AP2
kDa M 1 2 3 4 5 6
100-
70-
55-
40-
35-
25-
AZU1
kDa M 1 2 3 4 5 6
35-
25-
15-
10-
shorter exposure
PDI
kDa M 1 2 3 4 5 6
100-
70-
55-
40-
35-
25-
AZU1
kDa M 1 2 3 4 5 6
35-
25-
15-
10-
longer exposure

Supplementary Figure S2. Immunohistochemistry staining of early and late recurrent PDAC organoid cultures correlated to organoid source tumor biopsies. Original source tumor tissues and corresponding organoids were stained for HE, MAEA, NT5E, ATP62 and MICA. Non-tumor chronic pancreatitis served as control.

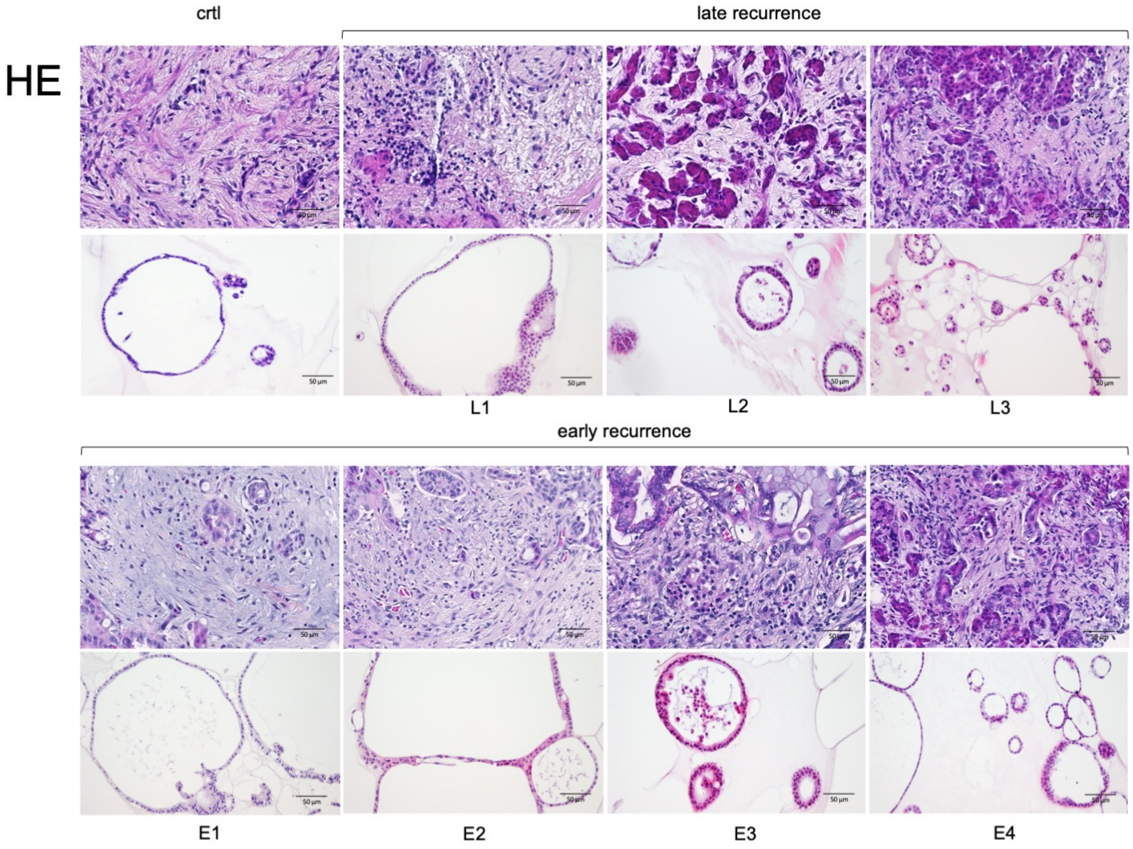

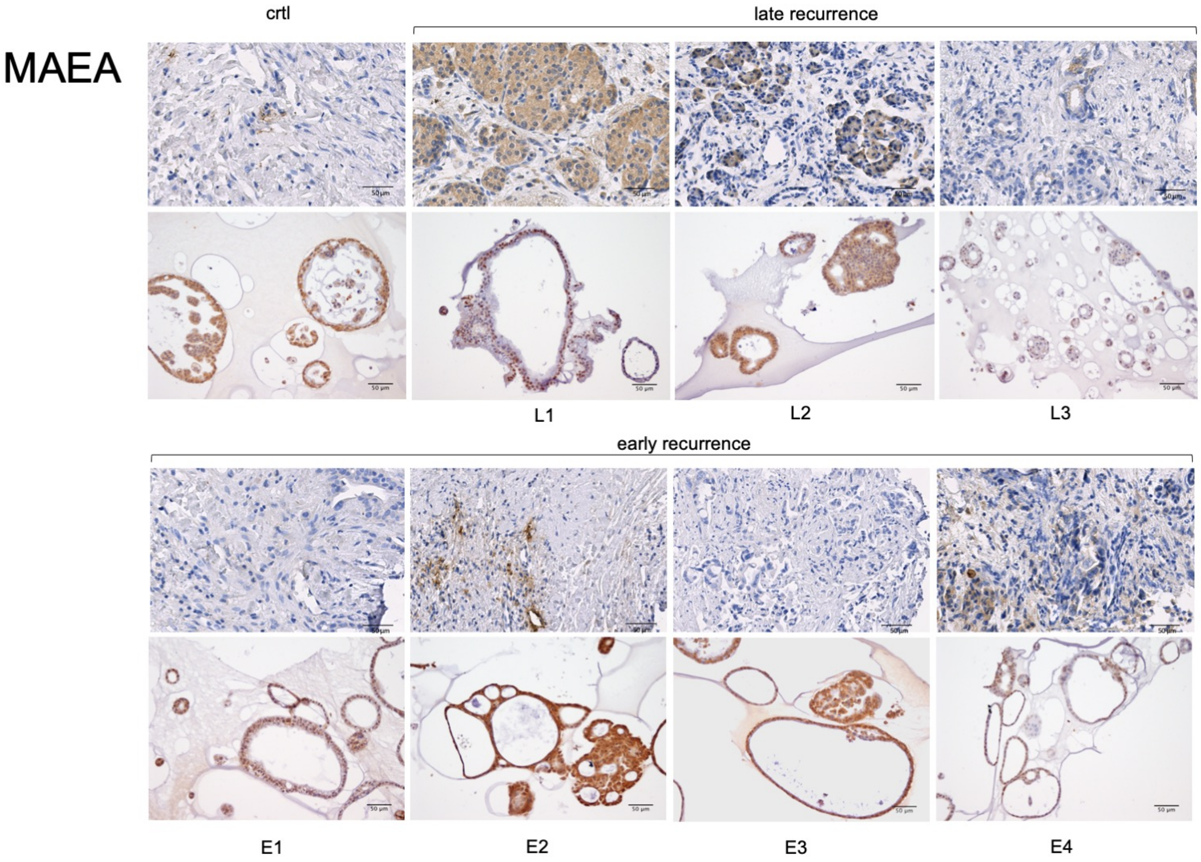
MAEA
crtl
late recurrence
L1
L2
L3
early recurrence
E1
E2
E3
E4

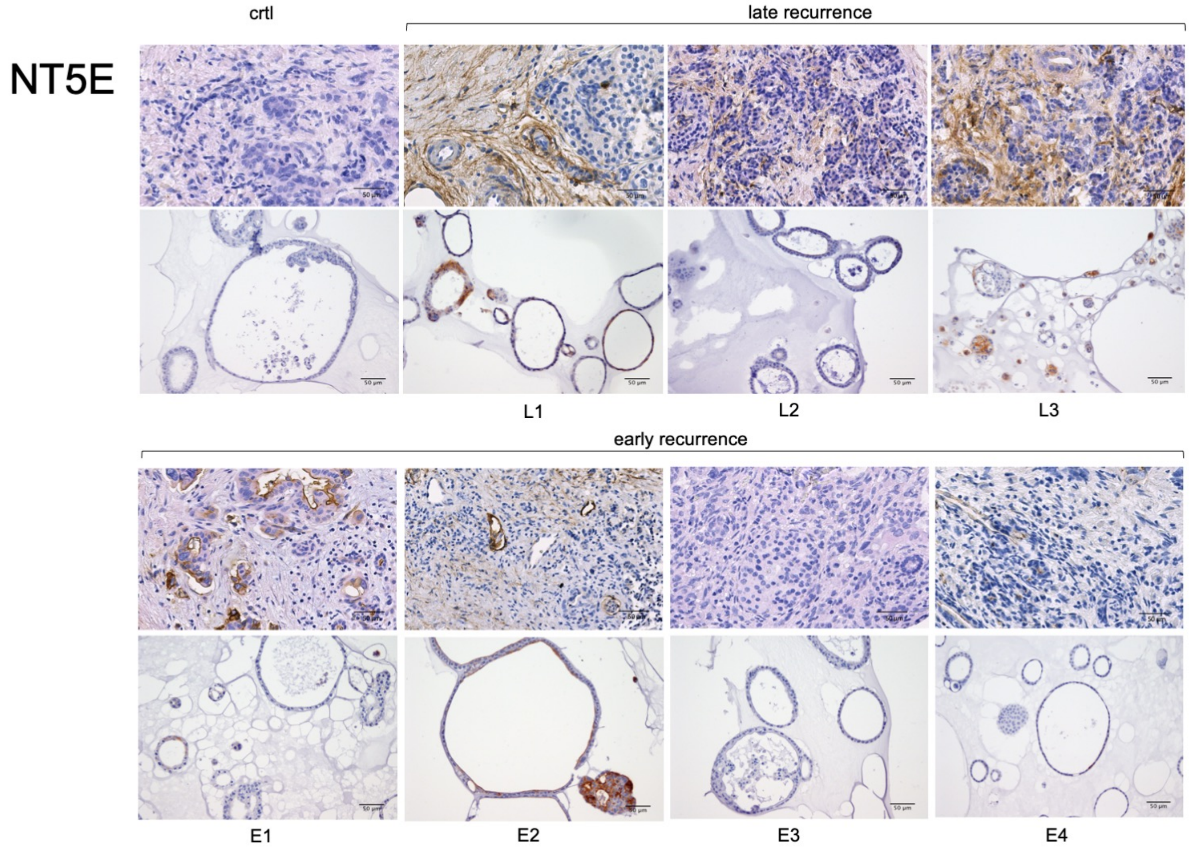
NT5E
crtl
late recurrence
L1
L2
L3
early recurrence
E1
E2
E3
E4

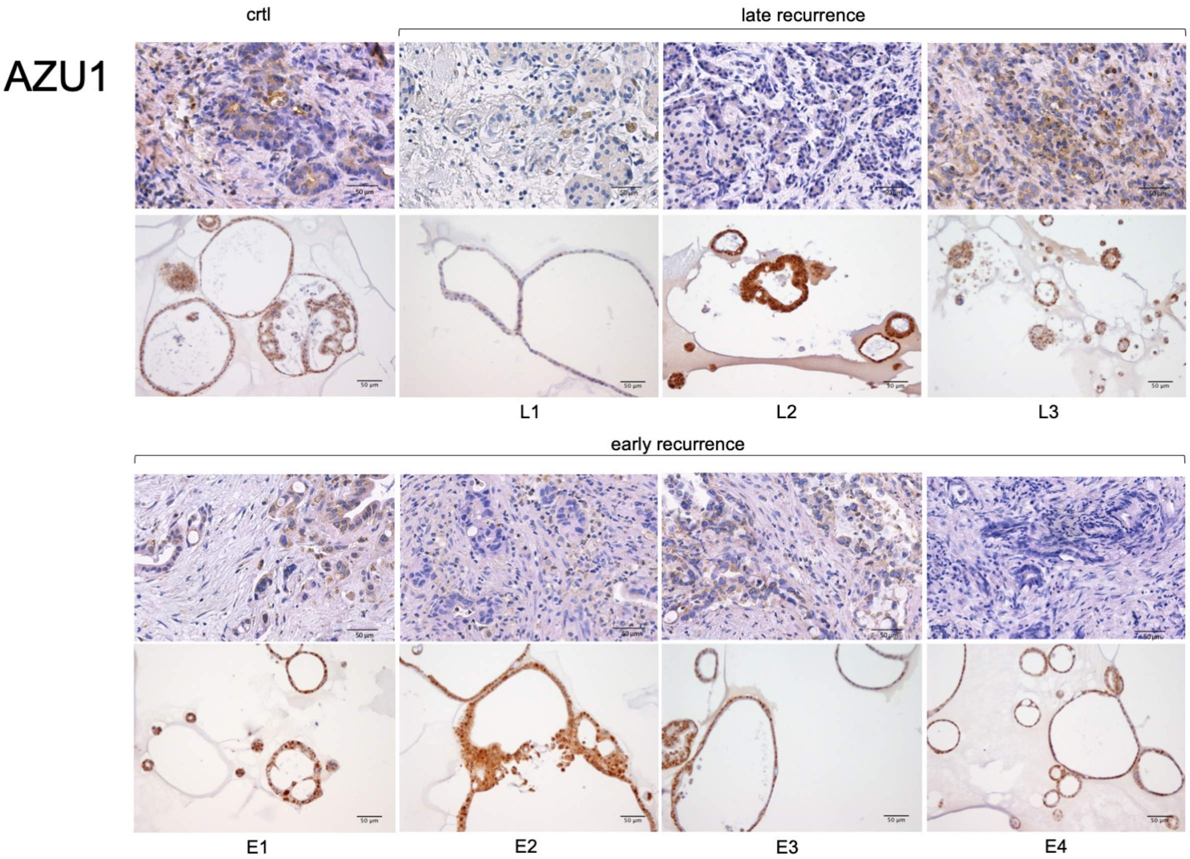
AZU1
crtl
late recurrence
L1
L2
L3
early recurrence
E1
E2
E3
E4

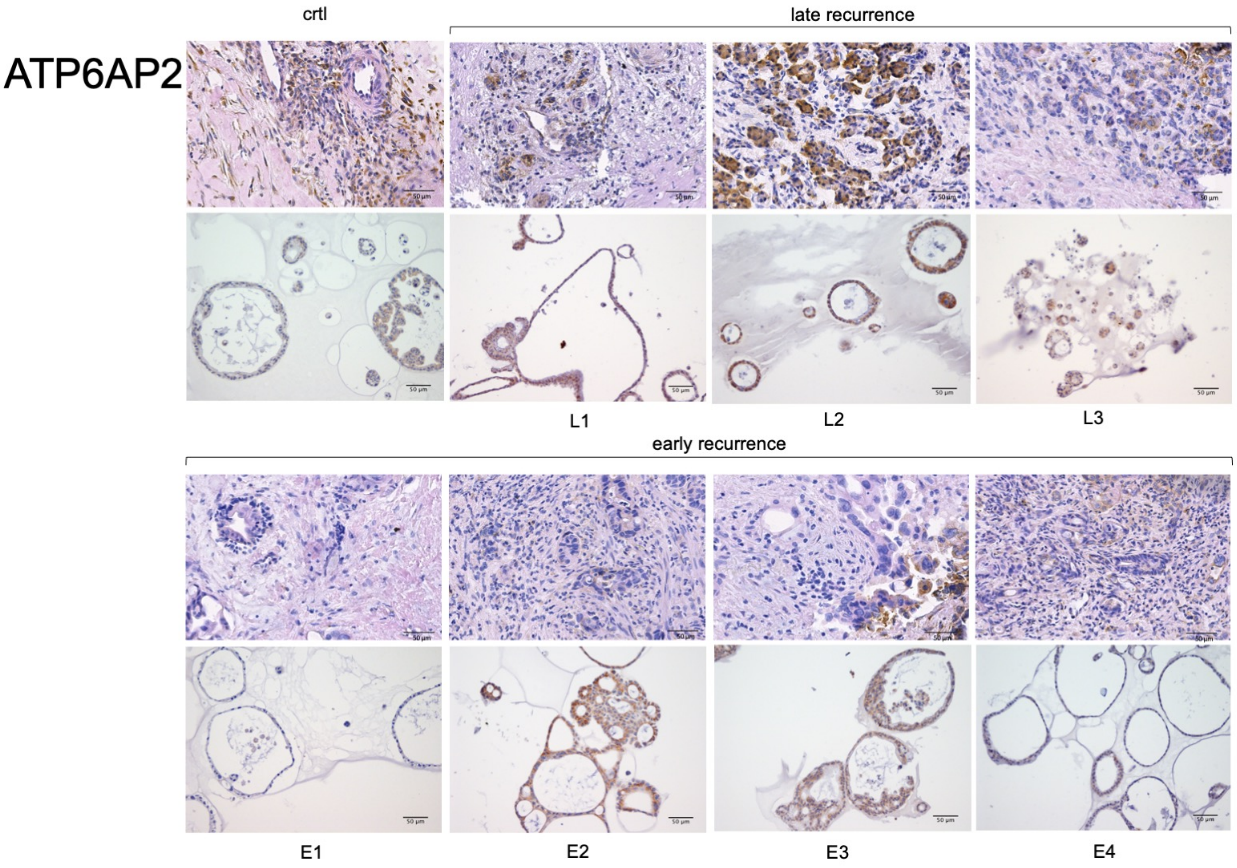
ATP6AP2
crtl
late recurrence
L1
L2
L3
early recurrence
E1
E2
E3
E4

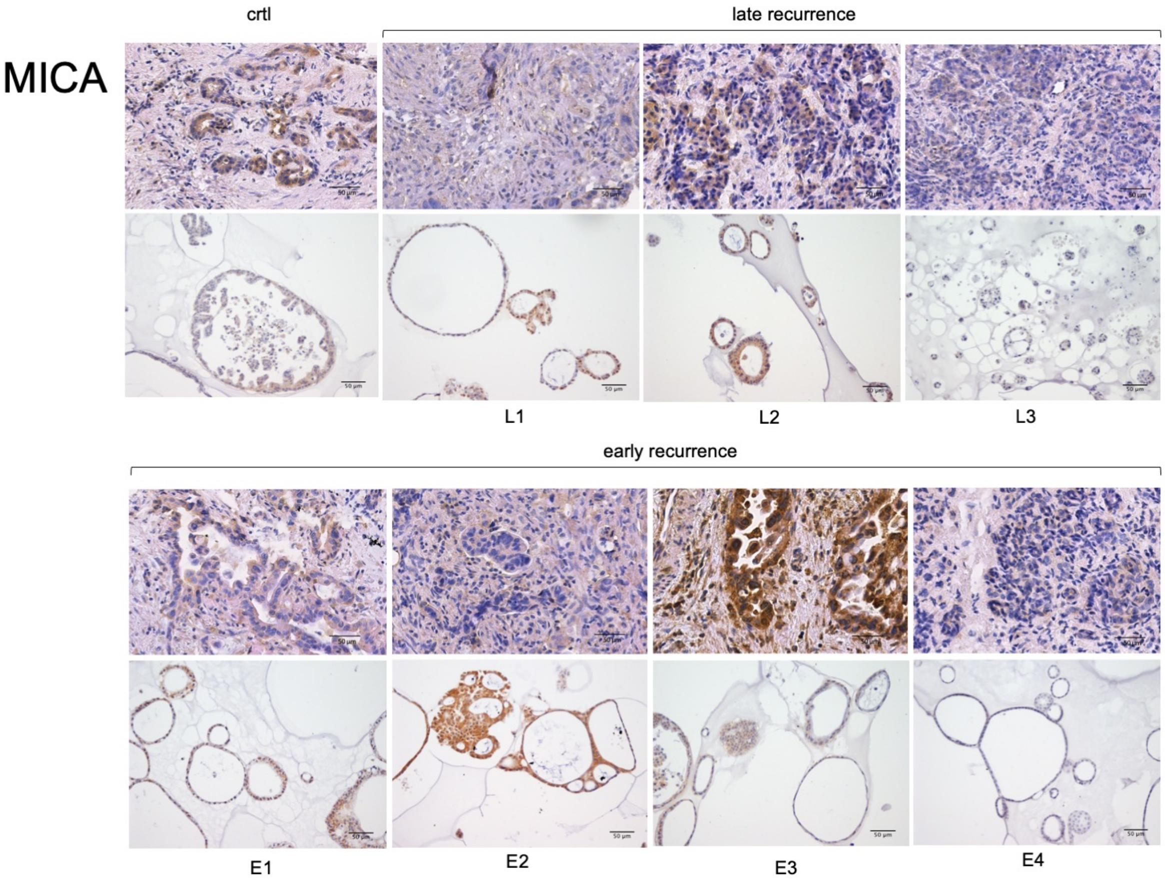
MICA
crtl
late recurrence
L1
L2
L3
early recurrence
E1
E2
E3
E4
50 μm
